# Dopamine D2 Receptor Isoform Heteroreceptor Complexes with the Growth Hormone Secretagogue Receptor 1a Reveals Isoform-Specific Interaction Interface Dynamics

**DOI:** 10.1021/acs.jcim.6c00308

**Published:** 2026-03-28

**Authors:** Álvaro Cáceres-Quezada, Dasiel O. Borroto-Escuela, Angélica Fierro

**Affiliations:** 1 Neurochemistry and Molecular Modeling Lab, Department of Organic Chemistry, Escuela de Química, Facultad de Química y de Farmacia, Pontificia Universidad Católica de Chile, Santiago 7820436, Chile; 2 Receptomics and Signaling Networks in Brain Diseases (Group C22), Instituto de Investigación Biomédica de Málaga y Plataforma en Nanomedicina−IBIMA Plataforma BIONAND, Málaga 29010, Spain; 3 Receptomics & Brain Disorders Lab, Department of Human Physiology and Physical Education and Sport Sciences, School of Medicine, University of Malaga, Málaga 29010, Spain; 4 Centro Interdisciplinario de Neurociencia Aplicada, 28033 Pontificia Universidad Católica de Chile, Santiago 7820436, Chile

## Abstract

The dopamine D2 receptor (D2R), a class A G-protein-coupled receptor expressed in two isoforms at the central nervous system, has been implicated in several neuropsychiatric and neurodegenerative disorders. Although conventionally understood as a monomer, D2R can exist as homo- and heteroreceptor complexes, with each structural state conferring distinct functional properties. The heteromer formed by the D2R and the growth hormone secretagogue receptor (GHSR1a) has been primarily studied in the context of eating disorders, but more recently, in Parkinson’s disease mouse models. Besides the neuropharmacological relevance of this heteromer, the molecular mechanisms underlying their interaction interface in complex formation have not been fully understood. Moreover, the specific contribution of each D2R isoform to both the formation and functional properties of the D2R/GHSR1a heteroreceptor complex remains to be elucidated. Therefore, in this study, we aimed to characterize the structural and dynamic differences between D2R short and long isoforms, both as monomers and within the D2R/GHSR1a heterocomplexes. Through computational methodologies including homology modeling, receptor–receptor docking, and coarse-grained molecular dynamic simulations, we observed distinct behaviors for D2R isoforms as monomers and in heteromeric assemblies with GHSR1a. Our findings showed differences in D2R isoform motions that may impact their activation along with isoform-specific interaction interfaces with GHSR1a. Furthermore, these differential interfaces promoted site-to-site and ligand-binding pocket differences for both D2R isoforms, suggesting isoform-specific allosteric regulation potentially mediated by GHSR1a within the D2R/GHSR1a heterocomplexes. Overall, our results highlight the isoform-dependent mechanisms within the D2R/GHSR1a heteromer that influence complex formation, ligand binding, and intracellular signaling, providing a framework for future therapeutic strategies targeting D2R/GHSR1a heteroreceptor complexes.

## Graphical abstract

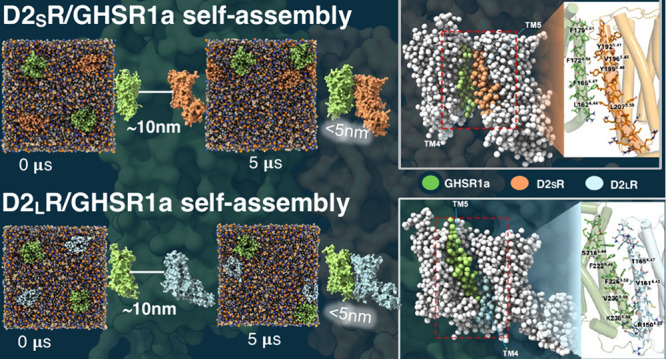

## 1 Introduction

Dopamine is a monoaminergic neuromodulator with important roles in the central nervous system (CNS) through long-range projections emerging from the two main dopaminergic nuclei, the ventral tegmental area (VTA) and the substantia nigra pars compacta (SNc), located at the midbrain.^1–3^ While engaging high-order functions to regulate physiological processes from cognitive to complex behavior in the brain,^4^ dysfunction of dopamine signaling has been related to several neurodegenerative and neuropsychiatric disorders including Parkinson’s disease (PD) and schizophrenia, among others.^5–8^

Dopaminergic signaling is mediated by the activation of five dopamine receptors (DRs): class A G-protein-coupled receptors (GPCRs) divided in two subfamilies as D1-like and D2-like DRs.^9^ They differ on the G-protein they bind to but also by their differential expression at the CNS. While D1-like receptors are located only postsynaptically in nondopaminergic cells, D2-like receptors are expressed both pre- and postsynaptically in different types of neurons.^10^ Presynaptically, dopamine 2 receptor (D2R) is found in both dopaminergic nuclei as an autoreceptor, modulating the excitability of axon terminals for the release of dopamine as an autofeedback inhibition process.^11–13^ Moreover, two isoforms of the receptor, which differ by 29 amino acids (residue 242 to 270) within the third intracellular loop (ICL3), are expressed at the CNS.^14,15^ The relevance of the ICL3 has been recently highlighted due to its position next to the effector-binding site of GPCRs and its connection with TMV and TMVI, which undergoes through relevant structural changes that condition the activation/inactivation states of the receptors.^16,17^ Several attempts to elucidate the specific roles for the short (D2_S_) and long (D2_L_) isoforms have suggested molecular and histological differences between them. Specially, their activation response upon agonist-binding and their cellular expression levels.^18–21^ Still, the relevance of the changes in length of the ICL3 for the functional differences of the isoforms is poorly understood.

Conventionally, class A GPCRs work as monomeric functional entities binding to an extracellular ligand that leads to conformational changes within the receptor to promote their respective downstream signaling cascades.^22,23^ However, conformational changes and signaling can be mediated by receptor–receptor interactions as well, functioning as homomeric and/or heteromeric macromolecules with different and novel functions.^24–26^ The D2R operates as a monomer but can also assemble into homodimers and diverse heteroreceptor complexes, many with significant clinical implications in neuropsychiatric and neurodegenerative disorders.^27–31^ For instance, the activation of the D2R protomer in mice produces an anorexigenic effect, based on the cabergoline dose-dependent suppression of food intake due to the activation of the D2R/GHSR1a heteroreceptor complex.^32^ Given the neuroanatomical distribution of GHSR1a in both dopaminergic nuclei,^33,34^ the D2R/GHSR1a heterocomplex is also relevant in the context of Parkinson’s disease (PD), with GHSR1a interacting with D2-autoreceptors at presynaptic terminals. The experimental evaluation of the role of the D2R/GHSR1a heteroreceptor complex in SNc dopaminergic neurons showed improvement of locomotor activity and promotion of synthesis and release of dopamine in a PD mouse model.^35^ However, despite the clinical relevance of D2R/GHSR1a heterocomplexes on PD and eating disorders, little is known about their molecular and structural–functional mechanisms underlying their formation.

Experimental and computational studies have focused on determine the existence of these oligomeric arrangements and the interaction interface of GPCR heteromer formation as well.^36–39^ Several reports for the interactome of D2R while forming homomeric and/or heteromeric complexes with other GPCRs have shown a possible relevant role of transmembrane domains (TMs) I, II, IV, and V at the interface.^40–42^ To date, insights on the biological relevance of the interaction interface in the heteromer formed between D2R and GHSR1a remain unclear. Moreover, given the structural difference for D2R isoforms, understanding the interaction mechanisms for the short and long isoform with GHSR1a might increase the knowledge regarding the physiology and altered states of dopaminergic signaling, both in neuropsychiatric and neurodegenerative diseases. In this study, we sought to characterize the isoform-specific differences in the interaction interface of D2R/GHSR1a heteroreceptor complexes through different in silico approaches, including receptor’s homology modeling, receptor–receptor docking, and coarse-grained dynamic simulations. Our analyses reveal intrinsic differences in the motions of the D2R isoforms, including distinct modulatory effects exerted by GHSR1a, apparently dependent on the transmembrane domains involved in the interaction interface.

## 2 Materials and Methods

### 2.1 Receptor Structures

The D2_S_R, D2_L_R, and GHSR1a homology model structures were obtained using the crystal structures available in the Protein Data Bank for the human D2R (PDB ID: 6CM4) and the human GHSR1a (PDB ID: 7F9Y). The selection criteria for the templates were based on the availability of the intra- and extracellular loops structures resolved, as well as the identity of the residues prior sequence alignment. Due to advantages in loop modeling and membrane macromolecule build,^43^ the Modeler program 10.4 ^44^ was used to perform 500 runs with the standard parameters and minimization was done using the optimizer module in the same version 10.4 of the program. Based on the lowest values for the atomic distance-dependent statistical potential (DOPE)^45^ scoring function, we further evaluated 10 models through geometric, stereochemical, and energetic criteria analysis using the ProSA^46^ and PROCHECK^47^ servers. Best models were selected based on energetic and stereochemical criteria. Although the full structure for each receptor was modeled, part of the N- and C-terminals for the three receptors were removed due to lack of structural information and difficulties to stabilize the systems in the MD simulations. Specifically, for the D2R isoforms, 32 residues were removed only from the N-terminal, and for the GHSR1a, 31 residues from the N-terminal and 30 residues from the C-terminal were removed.

### 2.2 Coarse-Grained Molecular Dynamics Simulations

The coarse-grained molecular dynamic (CG-MD) simulations were run in GROMACS v2021.4 ^48^ with the Martini 3 force field.^49^ *Martinize2* script^50^ was used in the coarse-grained topology generation for the all-atom models for the D2_S_R, D2_L_R, and GHSR1a while applying an elastic network (EN) restraint with elastic bond force constants of 700 kJ mol^–1^ nm^–2^ with a lower and upper cutoff of 0.5 and 0.9, respectively. Both the decay factor and decay power were set to 0. We employed an eight-component lipid membrane bilayer to represent the biological complexity of a brain plasma membrane, as proposed by Ingólfsson et al.^51^ Specifically, the outer leaflet was composed by 45% of cholesterol, 24% of phosphatidylcholine (PC) lipids (18% POPC and 6% PAPC), 11% of glucosylceramide (PNGS), and 10% of both phosphatidylethanolamine (PE) lipids (PAPE) and sphingomyelins (SM) lipids (DPSM). For the inner leaflet, the composition was 45% of cholesterol, 22% of PE lipids (PAPE), 15% of both PC lipids (POPC and PAPC) and phosphatidylserine (PS) lipids (PAPS), 2% of SM lipids (DPSM), and 1% of phosphatidylinositol (SAP2). *Insane-b8* script^52^ was used to construct the system box with the membrane bilayer and water molecules using the default parameters. For the self-assembly assays, in-house scripts were used to construct the “chess board” with two repeats of each protein per system as explained in Section 3. The protocol used for every system comprehends 2500 steps of steepest descent minimization based on default parameters of Martini, while applying position restraints in the *Z* axis and no constraints, allowing full relaxation of bonds and angles. Later, four equilibration steps with increasing time steps were employed (10 ps with a time step of 1 fs, 100 ps with a time step of 5 fs, 450 ps with a time step of 10 fs, and 20 ns with a time step of 20 fs), followed by a 5 μs production run, both in the NPT ensemble (*T*= 310.0 K; *P*= 1 atm) using Berendsenthermostat and barostat. The scripted parameters for minimization, equilibration, and production are available in the Data and Software Availability Statement section of the article. For each simulation system, three independent production CG-MD simulations were performed, having 15 μs total per system.

### 2.3 Trajectory Analysis

The root-mean-square deviation (RMSD) was calculated for the backbone atoms with respect to their positions at the reference structure (0 ns) by using the RMSD Trajectory Tool available in VMD.^53^ By using an in-house script in the tk console of VMD, root-mean-square fluctuation (RMSF) was calculated for the Cα atoms of each residue for the whole trajectory. For the HADDOCK heterodimers, the RMSF was calculated for both receptor protomers at the same time. The principal component analysis (PCA) was performed using the gmx covar and gmx anaeig utility toolkits of GROMACS,^54^ to calculate and diagonalize the covariance matrix for the trajectory of the D2R isoforms and to further analyze the eigenvectors obtained previously.

### 2.4 Backmapping

From each system, three frames from the coarse-grained molecular dynamic (CG-MD) simulations (initial, middle, and end frame; upon stabilization of the structures) were backmapped to CHARMM all-atom representation^55^ using the backward method through the initram interface, as previously described by Wassenaar et al.^56^ For the self-assembly assays, both receptor protomers were backmapped individually taken the frame where distance between receptor protomers was the lowest and the area of contact was the highest.

### 2.5 Protein–Protein Docking

The protein–protein structures for the D2_S_R/GHSR1a and D2_L_R/GHSR1a heteromers were obtained using the HADDOCK 2.4 server.^57,58^ Briefly, we followed ab initio docking method with some modifications. For the input parameters, we selected two active residues in each protein (Tyr192^5.41^ and Tyr199^5.48^ in D2R and Ser217^5.43^ and Ser218^5.44^ in GHSR1a). We then reduced the relative solvent accessibility (RSA) of the active residues to 5.0 and amplified to 15.0 Å (Å) the radius to define “passive residues” around the active ones. For the docking parameters, 50,000 structures were sampled for the first rigid body docking (it0), and then, 400 structures were sampled for the further semiflexible refinement and the latest final refinement (it1 and itw, respectively), as proposed by Koukos et al.^59^ The selection of the best receptor–receptor complexes was based on the position and *Z* angle of the receptors. Quantitative analysis of the average solvent accessible surface area (SASA) per residue was performed with the gmx sasa tool and adapted from the analysis performed by Di Marino et al.^60^

### 2.6 Self-Assembly Heteromer Evaluation

The obtained heteromers in the self-assembly assay, with differential interaction interfaces, were analyzed and compared using the PRODIGY^61^ and PRODIGY-CRYSTAL^62^ servers. Briefly, PRODIGY was used to quantitatively estimate the number of interfacial contacts (ICs), binding affinity (Δ*G*) of the complex, and its dissociation constant (*K*_d_). PRODIGY-CRYSTAL was used to determine the biological relevance of the interaction interface based on a trained machine learning algorithm.^63^ The suitable heteromers for both D2R isoforms were selected based on the lowest Δ*G*, lowest *K*_d_, and highest biological relevance.

### 2.7 Cavities and Contact Network Analysis

The cavity volume for the D2R isoforms as monomers and self-assembled heteromers with GHSR1a was performed using the CavitOmix plugin in Pymol.^64^ Briefly, based on the calculation for the physicochemical properties along with the potential interactions in the target protein structures, from all the possible cavities detected, we cleared those corresponding to the orthosteric binding pocket of D2R isoforms. The contact network analysis for the monomeric D2R isoforms and the self-assembled heteromers with GHSR1a was performed as previously described by Robles et al.^65^ Briefly, the contacts for each system were obtained using the RING server.^66^ To identify the site-to-site contact network, we first established the conservation of the interaction network within the orthosteric binding pocket (OBP) of both D2R isoforms based on the information for crystallographic structures available at the psnGPCRdb server.^67^ From these conserved amino acids, immediate neighboring residues in the contact network were selected and the site-to-site path was identified.

## 3 Results

### 3.1 Differences in the Monomeric Dynamics of D2R Isoforms

Due to the extensive sequence diversity, length, and intrinsic disorder of ICL3 among GCPRs, most of the current available crystallographic structures for GPCRs at the Protein Data Bank (PDB) lack this region. Therefore, to capture the relevance of ICL3 in the broad and coordinated conformational changes primarily for the D2R isoforms and GHSR1a, we performed homology modeling to solve the complete structure of the three receptors in study.

Based on the 500 models generated per receptor and the thorough analysis of 10 models per receptor as well, we selected the most suitable models (Figure S1) and performed CG-MD simulations to follow the broader conformational changes for all the GPCRs in study. The backmapped frames for the structures were used to compare the TM changes primarily between D2R isoforms, with a potential impact in the heteromerization with GHSR1a. A differential behavior was observed between the short and long isoform of D2R. The major displacement for the short D2R isoform (D2_S_R) was associated with TMI, TMVI, and TMVII (>6 Å in contrast to less than 4 Å displacement of the rest of TMs), in contrast with a conserved but global movement for the long D2R isoform (D2_L_R) (>4 Å for all the TMs displaced) (Figure 1A). Although both systems showed important changes at the ICL3, we observed a displacement of the D2_L_R-ICL3 away from the membrane (Figure 1A). Along with this visual evaluation, the root-mean-square deviation (RMSD) for the backbone of the receptors showed a global stability between 4 Å and 6 Å approximately for both systems (Figure S2A). For the root-mean-square fluctuation (RMSF) of the backbone, we noticed that the residues that fluctuate the most are primarily the ones forming the abovementioned ICL3, without significant magnitude (Å) differences between D2_S_R and D2_L_R (Figure S2A). There were no significant global changes (Figure S2B) in the TMs for GHSR1a, but an important loss of the secondary structure in a portion of the TMV was seen. Further evaluation of these behavior showed a change in the orientation of I219’s side chain that could be relevant in heteromerization (Figure S2C).

**Figure 1:**
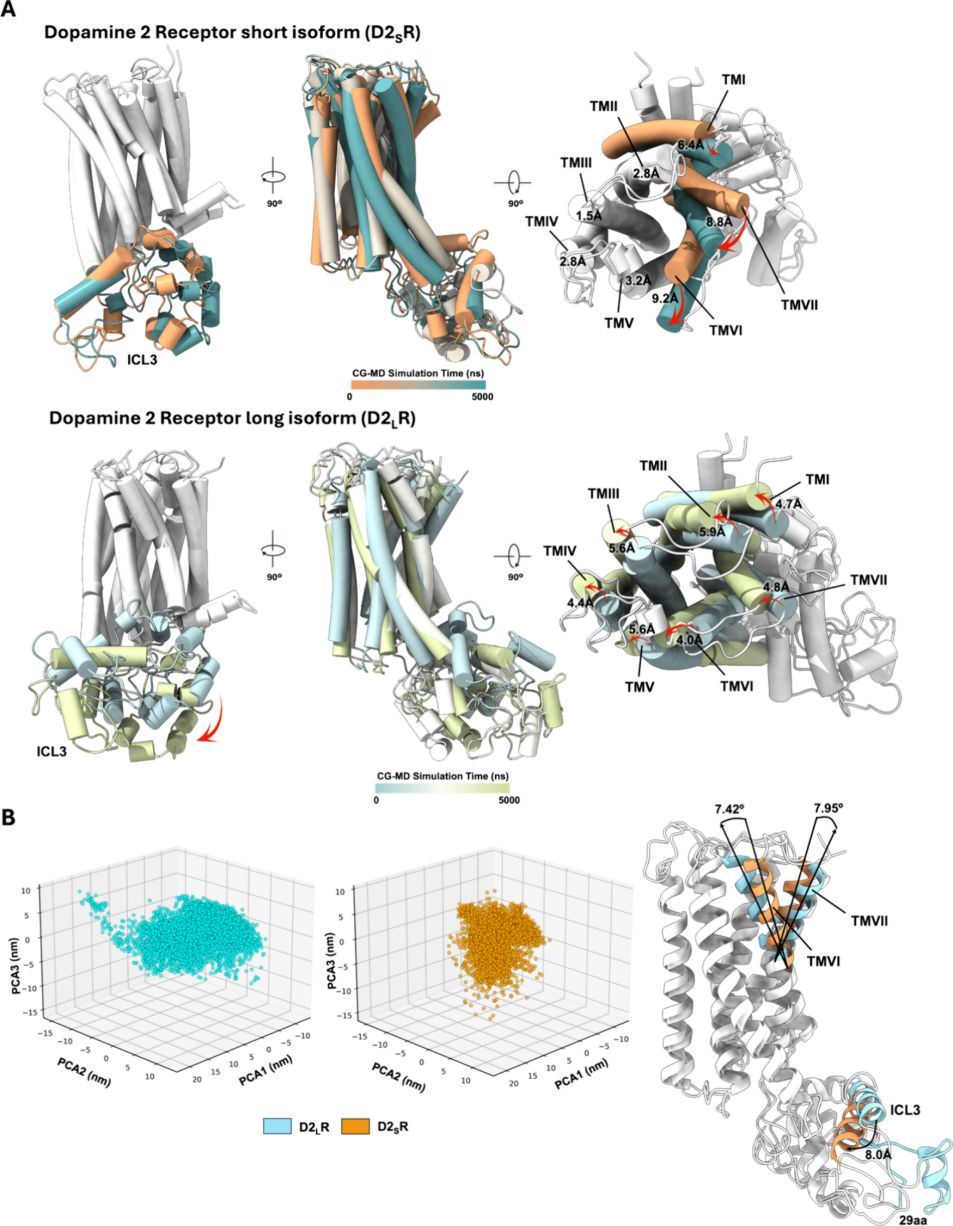
CG-MD simulations for D2_S_R and D2_L_R isoforms. (A) Three frames (initial, middle, and end frame) of CG-MD simulations. On the upper part, D2_S_R dynamic shows displacement of TMI, TMVI, and TMVII (red arrows). On the lower part, D2_L_R dynamic shows global displacement of all the TMs and ICL3 as well (red arrows). Both receptors dynamics were in the apo state. (B) 3D projection of the principal component analysis (PCA) shows broader conformational changes for D2_L_R (light blue) in contrast with D2_S_R (orange). Superposition of D2R isoforms shows differences in TMVI and TMVII, along with the ICL3 between them.

To further evaluate if there are differences between the D2R isoforms, we performed a principal component analysis (PCA) aiming to capture only the most relevant conformational changes in both systems during the dynamic simulations. PCA results allowed us to observe common points of interaction between D2R isoforms, but important differences in terms of broader conformational changes specifically for the long isoform of D2R are relevant (Figure 1B), primarily observed at the upper portion of TMVI and TMVII, along with ICL3 differences.

### 3.2 Stability and Residue Difference Motion in a Biased Heteromer Interface

Given the absence of molecular information regarding the important residues that promote the interaction interface at the hD2_S,L_R/hGHSR1a heteromers, we next based our analysis on several studies for oligomeric macromolecules for D2R.^68,69^ Knowing the relevance of the TMIV and TMV, we conducted our heteromeric complex formation through a biased protein–protein docking using the HADDOCK 2.4 server. HADDOCK scoring involves a linear combination of 13 terms (VDW, Coulombic, among others), which describes a protein–protein complex through three global terms: (i) HADDOCKscore-it0 (rigid body), (ii) HADDOCKscore-it1 (semiflexible), and (iii) HADDOCKscore-it2 (explicit solvent refinement). As described in ref 27, while selecting Tyr192^5.41^ and Tyr199^5.48^ in D2R and Ser217^5.43^ and Ser218^5.44^ in GHSR1a as “active” residues guiding a TMV/TMV interaction interface, we obtained a suitable heterodimer that was further evaluated in CG-MD simulations, primarily to determine the stability of the structure in time. Both systems showed an interaction interface through TMIV and TMV that seems stable along the 5 μs CG-MD simulations (Figure 2A), also observed by the RMSD for the backbone of the heteromers, with a global stability between 4 Å and 8 Å (Figure S3A). Additionally, the RMSF of their backbones showed primarily the fluctuation of residues associated with ICL3 from both receptors and in both systems (Figure S3B).

**Figure 2:**
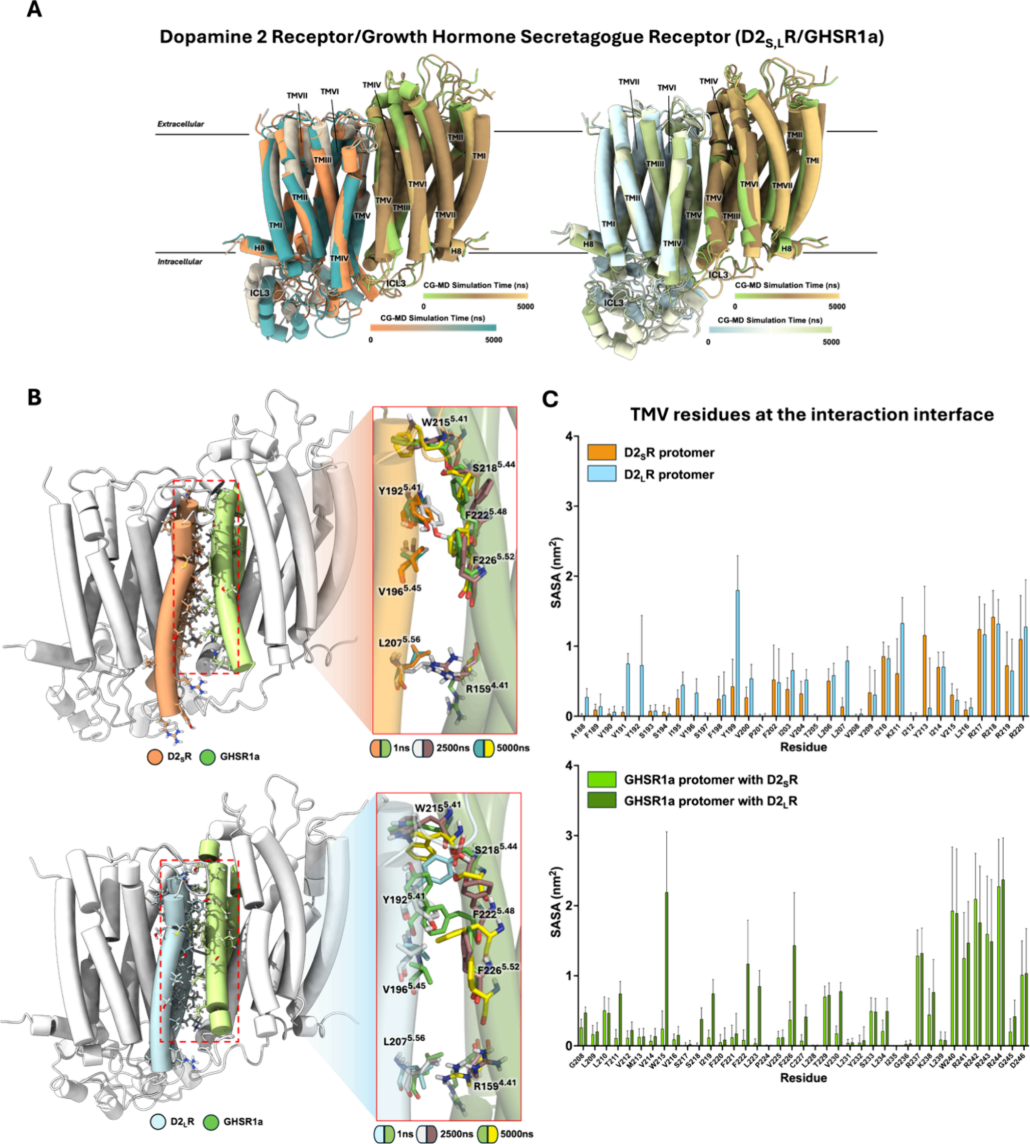
CG-MD simulations for D2_S,L_R/hGHSR1a heteromers. (A) Three frames (initial, middle, and end frame) from one of the 5 μs CG-MD simulations for the heterodimers obtained in HADDOCK. The D2_S,L_R/hGHSR1a dynamics show global stability with an interaction interface by TMIV/V from both receptors (D2sR in orange and D2_L_R in light blue). (B) Interaction interface at the three frames (initial, middle, and end frame) from one of the 5 μs CG-MD simulations. Differences in aromatic side chains motions for D2_L_R/hGHSR1a are relevant to follow (lower part). (C) Average solvent accessible surface area (SASA, nm^2^) for all the residues from TMV at the interaction interface for D2R isoforms and GHSR1a protomers. Differences in nonpolar aliphatic and aromatic residues could be observed.

To further understand the stability of the heteromer formed by D2R isoforms with GHSR1a, we used the PRODIGY server, aimed to predict the main residues in contact at the interaction interface for the heteromer obtained by the HADDOCK server. Seventy-nine contacts were predicted along the TMIV and TMV primarily (Table 1), some of them correlating with the abovementioned important residues for the D2R interaction. We focused the analysis on those residues from both TMs interacting to see changes in angle and position during the dynamics. Although both showed pi-aromatic interactions at the interface, the interactome for D2_L_R showed differences in motion during the dynamic simulations (Figure 2B). This was further evaluated quantitatively by calculating the average SASA of the residues implicated at the interaction interface for both D2R isoforms and GHSR1a during the 5 μs CG-MD simulations. Specifically, differences in some nonpolar aliphatic (V191^5.40^, V196^5.45^, and L207^5.56^) and aromatic (Y192^5.41^, Y199^5.48^, and Y213^5.62^) residues at the TMV between the short and long isoforms of the D2R could be observed (Figure 2C). Moreover, differences at the GHSR1a protomer residues (nonpolar aliphatic residues such as I219^5.45^, L223^5.49^, and V230^5.56^ and aromatic residues such as W215^5.41^, F222^5.48^, and F226^5.52^) were also observed regarding the D2R isoform it was interacting with (Figure 2C).

**Table 1:** PRODIGY Protein–Protein Quantitative Interaction Interface Analysis^a^

protein–protein complex	binding affinity (Δ*G*, kcal/mol)	dissociation constant (K_d_, M)	ICs charged-X*	ICs polar-X*	ICs nonpolar–nonpolar	predicted interface (biological/crystallographic)
D2_S,L_R/GHSR1a TMV/TMV (HadDock dimer)	–9.3	1.5 · 10^–7^	17	18	44	0.92/0.08
D2_S_R/GHSR1a TMI-TMIV	–5.1	1.8 · 10^–4^	15	1	5	0.272/0.728
D2_S_R/GHSR1a TMV-TMIV	–5.9	4.8 · 10^–5^	8	11	41	0.824/0.176
D2_L_R/GHSR1a TMI-TMIV	–6.8	9.7 · 10^–6^	19	8	37	0.856/0.144
D2_L_R/GHSR1a TMIV-TMV	–9.0	2.6 · 10^–7^	40	15	8	0.3/0.7

a X = Any residue charged, polar, or nonpolar. ICs: Interfacial contacts.

### 3.3 Stability and Differential Interaction Interface for D2R Isoforms in Unbiased Heteromer Formation

Given that the HADDOCK server allowed us to select the main “active” residues for the interaction interface of both heteromeric complexes, we wanted to certainly evaluate the interaction mode of the receptors in a dynamic process, also to observe if there is any difference in the interaction between D2R isoforms with GHSR1a. While working with the CG-MD simulations, we worked with self-assembly simulations to characterize the receptor–receptor interaction interfaces by creating supramolecular high-order architectures from the organization of individual proteins in a membrane patch.^70,71^

To simplify the complexity of a biological membrane, we constructed lipid bilayer patches for each receptor, two monomers of each D2R isoform and two monomers of GHSR1a, distributed randomly (Figure 3A). We followed the formation of homomeric and/or heteromeric complexes, but only heteromers were formed between the D2R isoforms and GHSR1a. The interaction of receptors was determined by a decrease in the distance between the center of masses of the proteins and the increment in contact between them. When the distance between their center of mass was lower than 5 nm and the area of contact was closer to 20 nm^2^, the receptors were interacting (Figure 3B). Interestingly, two different interaction modes were obtained for the D2_S_R self-assembly system, involving TMI/TMIV and TMV/TMIV from D2_S_R and GHSR1a, respectively (Figure S4A). Likewise, two interaction modes were also obtained for the D2_L_R self-assembly system, but TMI/TMIV and TMIV/TMV from D2_L_R and GHSR1a were involved, respectively (Figure S4B). We used the PRODIGY server to quantitatively determine the best heterodimer and to determine the biological relevance of the interaction interface as well.

**Figure 3:**
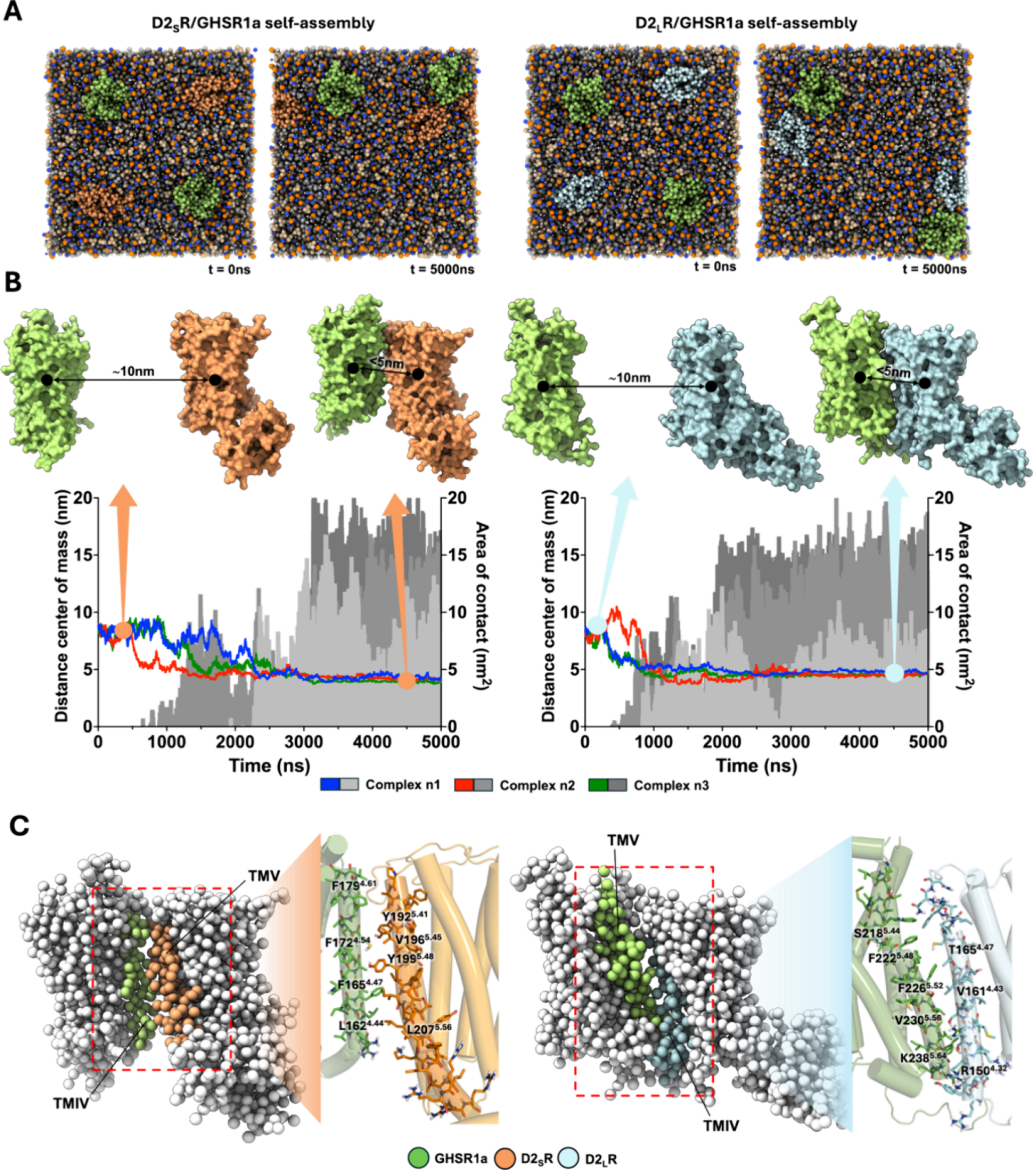
Self-assembly CG-MD simulations for D2R isoforms with GHSR1a. (A) Representation of the self-assembly system for both systems. Initial frame (0 ns) shows the random distribution of D2R and GHSR1a in each membrane patch. A final frame (5000 ns) shows the heteromers formed (top right for D2_S_R self-assembly and top left for D2_L_R self-assembly). (B) Graphs are for three complexes from three independent 5 μs CG-MD simulations. When distance between monomers decreases shorter than 5 Å, the area of contact starts to increase. The interaction in all the structures is stable for at least 2.5 μs. (C) Representative heteromers D2_S,L_R/GHSR1a with differential TMIV/V interaction interface. The contacts were evaluated in the backmapped all-atom structures, and the main residues are highlighted at the interface.

For both heteromeric complexes with each D2R isoform, the TMIV/TMV interaction interface seems to be better quantitatively considering three out of the four parameters previously mentioned (Table 1). We then isolated the CG heteromeric structures formed by TMIV/TMV interactions at the CG-MD simulations, and we studied the residues of each TM participating at the interface by backmapping them. Based on the contacts previously obtained from the PRODIGY server, we observed pi-aromatic interactions at the interface of both heteromers and the differences between each other due to the differential TMs forming the interface, as previously commented (Figure 3C).

### 3.4 Differences in Cavities between D2R Isoforms as Monomers and at Heteromeric Complexes

Following an allosteric theory where GHSR1a modifies the properties of D2R, we evaluated the differences in OBP’s cavity volume between monomeric and protomeric D2R isoforms, which might be promoted by the GHSR1a allosteric regulation. While the cavities volume changed in both D2R isoforms when forming the heteromer with GHSR1a, we observed a reduction for the cavity’s volume in the short isoform of D2R in the heteromer (Figure 4A, left) due to an inward displacement of 5.5 Å in the upper portion of the TMVI (data not shown), without significant changes in the charge of the OBP, which might not affect the availability of the protein to interact with the ligand dopamine at this region (Figure S5A). On the other hand, the volume of the cavity for the long isoform of D2R increased (Figure 4A, right) primarily due to a 2.1 Å broader distance between TMIII and TMVI also at the upper portion of the receptor when forming the heteromer with GHSR1a (data not shown). As well as for D2_S_R, the net negative charge of the OBP was not modified by the heteromer formation, which may not affect the receptor’s capability to bind to dopamine (Figure S5A).

**Figure 4:**
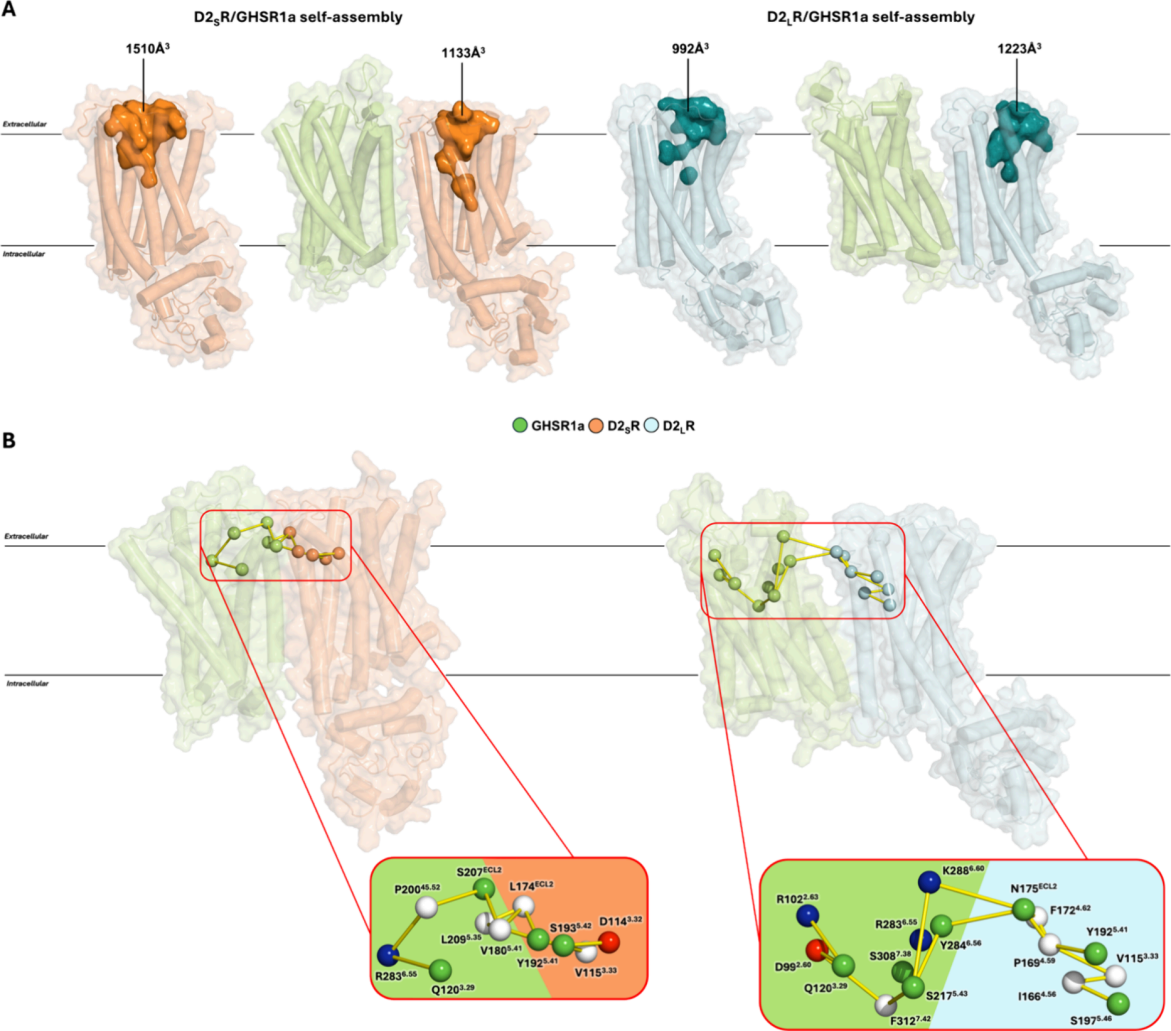
Cavities volume analysis and contact network for self-assembly D2_S,L_R/hGHSR1a heteromers.(A) Final frame of D2R isoform monomeric 5 μs CG-MD simulations and self-assembly D2_S,L_R/hGHSR1a heteromers show differences in cavities volume due to TMs displacements. (B) Differential site-to-site contact network. Cα atoms in spheres colored by amino acidic nature (red = acid, blue = basic, green = polar without charge, and white = nonpolar).

Later, the analysis was structured around the contact network for those residues connecting the respective orthosteric binding pocket (OBP) of the D2R isoforms with GHSR1a while forming the heteromers. For both heteromers in study, we were able to observe a contact network that communicates their OBPs (Figure 4B). Nevertheless, given the differences in the TM interaction interfaces, the site-to-site connecting network is different between the D2R isoform’s heteromers. On the one hand, for the short D2R isoform heteromer, we observed contacts based mainly on nonpolar–nonpolar interactions between hydrophobic residues (Figure 4B, left). On the other hand, the long D2R isoform interacted with GHSR1a by polar–polar interactions also with a strong presence of charged residues in GHSR1a OBP’s proximity (Figure 4B, right). Furthermore, given this difference in the site-to-site connecting network, we followed the changes in the OBP’s contact network for the D2R isoforms as monomers and also while forming the heteromer structure with GHSR1a. Interestingly, while the short D2R isoform presented more interactions in their OBP’s contact network in contrast with D2_L_R, both D2R isoforms expand their respective contact networks at the OBP while forming the heteromer with GHSR1a (Figure S5B). Besides the differences in the amount and nature of the residues in contact, this increment for the connecting network implies that the allosteric regulation of GHSR1a may help stabilize D2R ligands more effectively when forming the complex.

The differences in the cavity’s volumes and contact networks between the monomeric D2R isoforms with the ones forming the respective heteromer might be explained due to changes in the orientation of the side chains of these residues, having a special impact in the “activation/inactivation switches”. Further analysis showed that at least one residue of each “activation” motif presented differences between monomeric and heteromeric D2_S_R isoform, reinforcing an important role of GHSR1a in the regulation of the activity of the receptor (Figure S5C). For the D2_L_R isoform, we observed a global deviation of the structure in their TMs without affecting the orientation angle of the side chains in the “activation” motifs in study (Figure S5C).

## 4 Discussion

GPCRs are the largest subfamily of surface receptors coded by the human genome, where nearly 30% of the approved pharmacological treatments by the US Food and Drug Administration (FDA) are guided to a small fraction of them, highlighting their relevance in pathologic phenomenon.^72,73^ With the advances in crystallographic resolution of membrane receptors, several human D2R and GHSR1a structures have been resolved experimentally.^74–78^ This knowledge has promoted the study of class A GPCRs conserved structural rearrangements of residues and relevant motions in their TMs, nowadays well-known as “activation/inactivation switches”. These conformational changes are thought to start from the extracellular portion of the protein, throughout the TM-coordinated movement, and up to the cytosolic regions of the receptor.^79,80^ Nevertheless, flexible regions with significant relevance both to enhance the selective recognition of G-protein^81,82^ and promote receptor–receptor dimerization^83,84^ like the ICL3 are hard to resolve by crystallographic techniques and, so, difficult to study. The apparent distinct signaling properties between the two isoforms of the D2R expressed in the brain might be related to their ICL3 differential length, but little is known regarding the molecular mechanisms underlying their differential signaling states, as well as its influence in the physiological function of D2R isoforms in response to drug treatments.^85–87^ In this work, we have modeled the whole monomeric structure of the short and long isoform of D2R, along with GHSR1a, the three receptors in study. Although other methods such as ab initio loop modeling could have been used to differentiate the ICL3 specifically for D2R isoforms, full homology modeling was performed considering the nearly 150 amino acid residues forming the loop. Currently, ab initio methods are able to generate stable loop conformations in between 12 to 30 residues, while longer loops (>45 amino acid residues) have difficulties to predict reliable energetic and steric parameters for the structure.^88,89^ After performing CG-MD simulations of each monomeric receptor, important conformational differences were observed between the human D2R isoforms in an apo state. Furthermore, specific motions of the TMI, TMVI, and TMVII were relevant for the short isoform of D2R without significant differences in the intrinsic conformation of the ICL3. In contrast, the long isoform of D2R probed a global motion behavior while also promoting intrinsic structural disorganization of its ICL3 with motions toward the intracellular space (Figure 1A). Further analysis between both isoforms probed the broader conformational changes for the long isoform of D2R, with differences associated with changes primarily in the upper portion of TMVI and TMVII, some regions of the ICL3, and the 29 amino acids difference within this loop, according to the overlap of the average structures of the PCA results (Figure 1B). For the GHSR1a, the notable change so far, CG-MD simulations and PCA analysis of the D2R isoforms demonstrate differential global conformational changes between them. Also, CG-MD simulations showed the tilt angle of the TMV in GHSR1a due to the loss of the secondary structure for the I219’s side chain. Altogether, those differences and changes might produce an impact in the molecular properties of the receptors, affecting their heteromeric complex’s formation.

Given the raising knowledge of the homomeric and/or heteromeric formation capacity of these receptors from the past two decades, the rapid development of computational biochemistry has prompted the understanding of the molecular mechanisms underlying interaction interfaces between GPCRs, for the rational design of new pharmacological treatments relevant in neuropsychiatric and neurodegenerative disorders.^90–93^ In this work, we have reported for the first time a differential interaction interface for the D2R isoforms while forming a heteromer with GHSR1a. Although there exists experimental evaluation for the D2R/GHSR1a heteromer in SNc dopaminergic neurons,^35^ the description for the interaction interface within the D2R isoforms complex with GHSR1a (also based on the debatable pre- and postsynaptic expression pattern of D2R isoforms^94^) has not been explored so far. By using the HADDOCK server to obtain suitable heteromeric models for D2_S_R/GHSR1a and D2_L_R/GHSR1a based on its capability of producing acceptable and reliable membrane protein–protein dockings,^95^ we could observe that both heteromeric complexes are stable while interacting by their respective TMIV and TMV as an interaction interface, and specific differences in residue motions at the interface were observed for the D2_L_R/GHSR1a heteromer. Nevertheless, they were not able to disrupt the stability of the macromolecular structure. Given different reports for heteromerization of D2R with other class A GPCRs through their TMIV/TMV,^27,96^ as well as the implications that the Tyr199^5.48^ modifications has in the alteration for the oligomerization of D2_L_R;^97^ we thought to select it as one of the “active” residues in D2R to direct the heteromer’s formation into a suitable structure. However, due to the changes in the residue’s side chains at the interaction interface between D2R isoforms, specifically for the long isoform (Figure 2B), we thought to find a methodology that would allow us to determine the preferential zones of contact between the GPCRs in study without heteromerization bias, looking for a possible differential interaction interface for D2R isoforms.

By using large-scale CG-MD simulations, we were able to determine receptor–receptor interfaces in membrane bilayers with self-assembly assays.^90^ Although heteromerization was observed in every simulation (Figure 3B), we obtained two different interaction interfaces. In both heteromers, the TMIV/TMV interface probed to be the biologically relevant interface for the D2_S_R-GHSR1a heteromer but not for the D2_L_R-GHSR1a heteromer by the PRODIGY server (Table 1). This may be explained due to the high probability of interaction through the GHSR1a-ICL3, supporting a possible “domain-contact” heteromerization^83^ that needs to be further evaluated. Complex formation evaluation with self-assembly assays has been previously contrasted by applying docking assay for transmembrane components (DAFT) using Martini force field. Although several interaction interfaces were observed for the rhodopsin receptor homomerization, the TMIV and TMV interfaces highlighted their relevance in this GCPR interaction.^98^ Interestingly, we also observed an heteromer’s time formation difference between D2R isoforms, where the D2_L_R/GHSR1a complex is formed almost 1.5 μs faster than the D2_S_R/GHSR1a heteromer in all three cases (Figure 3B). By following the distances between TMs, interaction interfaces in GPCRs have been seen to go through local association and dissociation states before forming a “stable” oligomeric structure.^99^ Furthermore, time differences in the interaction interfaces and frequency of complex formation have been previously shown in self-assembly assays for monoaminergic transporters,^100,101^ but to our extent, this is the first report focusing on a differential interaction interface and timing for the heteromeric formation of D2R isoforms among the class A GPCRs family.

Allosteric modulation within GPCRs due to receptor–receptor interactions relates to the capability of one GPCR to modify the ligand-binding properties and/or the signal switching of the adjacent receptor while forming an heteromeric complex.^102,103^ Following an allosteric theory where GHSR1a modifies the properties of D2R, changing the native intracellular signaling pathway upon ligand binding,^104,105^ we aimed to analyze the CG-MD simulations to follow the changes of the important switches reported previously mentioned for class A GPCRs signaling, between the monomeric and heteromeric structures. Although proven experimentally for the D2R interaction with other class A GPCRs such as GHSR1a,^32^ the molecular changes that this receptor can lead to, specially at the OBP of D2R isoforms, had not been seen yet. While changes in the volume of the OBP are seen between the monomeric and heteromeric D2R isoforms, differences in the site-to-site contact network for each complex can also be observed (Figure 4). The increment in the number of contacts in the OBP of the D2R isoforms forming the complex (Figure S5B) could lead to an enhanced ligand stabilization that might promote changes in the intracellular signaling pathways as previously observed experimentally for the D1R/D2R heteromer.^106^ This signaling changes can also be related to changes in the residue’s motions along the receptors. Nevertheless, the vast majority of “activation/inactivation switches” and contact network studies available are based in crystallographic structures analysis, along with a comparison between monomeric proteins.^107^ A thorough analysis of class A GPCRs demonstrated common rotations, tilting, and/or switching of residue’s side chains that create contact networks to promote transitions between activation–inactivation states of these receptors. At the macroswitches scale, the most common change refers to the displacement of TMVI both extra and intracellularly. As for microswitches, the main contacts are observed between TMIII residues with TMV, TMVI, and TMVII side chains.^107^ Here, we observed that together with the increment in contacts at both D2R isoforms, the changes in the orientation of residue’s side chains at “activation” motifs for heteromeric D2R isoforms seems to be relevant for its inactivation/activation pattern (Figure S5C), altogether needs to be further evaluated in the presence of the endogenous ligand dopamine. Although the analysis is based on the allosteric modulation of GHSR1a toward D2R, we cannot discard the fact that D2R isoforms might exert a modulatory effect toward GHSR1a. Interestingly, while following the OBP’s interaction network for GHSR1a, the number of contacts was also increased in the heteromeric complexes formed (data not shown) promoting a similar effect that goes to both sides. Altogether, these results suggest differences between the monomeric and heteromeric D2R isoforms that can be related to the GHSR1a allosteric modulation of the D2R. To our extent, these results correspond to the first report correlating the changes of the contact network for the coordinated changes from OBP to “activation/inactivation switches” due to heteromerization of D2R with GHSR1a. The allosteric modulation of D2R toward GHSR1a cannot be discarded and could be further evaluated.

Overall, this study presents an in silico approach for the interaction interface in the heteromerization of the D2R isoforms with GHSR1a. We showed the intrinsic differences between D2R isoforms as monomers that suggested the demonstrated differences at the interaction interfaces of both heteromeric complexes formed as well as for their respective site-to-site contact networks. The CG-MD simulations allow us to gain insights into GPCR interactions, specifically for the D2R/GHSR1a heterocomplex, with a clinical relevance as new drug target for the treatment of PD, along with other neuropsyquiatric or neurodegenerative diseases.

## Supplementary Material



## Data Availability

All the main files to reproduce de CG-MD simulations for every system in study are publicly available at 10.5281/zenodo.17428514. The data set includes the initial structures of the modeled human receptors (D2_S_R, D2_L_R, and GHSR1a) in all-atom and coarse-grained resolution as monomers, the heteromeric structures obtained in HADDOCK, and the self-assembly arrangements used for the coarse-grained molecular dynamic (CG-MD) simulations. Also, folders with the topology for each receptor and the already prepared systems and all the CG-MD simulations parameter files are included as well.

## Notes

D2_S_R, D2_L_R, and GHSR1a model analysis and validation (Figure S1), trajectory analysis for D2_S_R, D2_L_R, and GHSR1a (Figure S2), the D2R isoform heteromers obtained from HADDOCK (Figure S3), interaction interface analysis for self-assembly assays output (Figure S4), and activation/inactivation switches analysis between monomeric and heteromeric D2R isoforms (Figure S5) (PDF)

## Abbreviations

CG-MD: coarse-grained molecular dynamics
D2R: dopamine D2 receptor
GHSR1a: growth hormone secretagogue receptor 1a
GPCR: G-protein-coupled receptor
OBP: orthosteric binding pocket
TMs: transmembrane domains
