# Supplementary material for: Dopamine D2 Receptor Isoform Heteroreceptor Complexes with the Growth Hormone Secretagogue Receptor 1a Reveals Isoform-Specific Interaction Interface Dynamics
Source: J Chem Inf Model. 2026 Mar 28;66(7):4174–86. doi: 10.1021/acs.jcim.6c00308 (PMC13080959; doi:10.1021/acs.jcim.6c00308)
Supplement: Supplementary file 1 [file ci6c00308_si_001.pdf]

Electronic Supporting Information

Dopamine D2 Receptor isoforms heteroreceptor complexes with the Growth Hormone Secretagogue Receptor 1a reveals isoform-specific interaction interface dynamics

Álvaro Cáceres-Quezada[1,2], Dasiel O. Borroto-Escuela[2,3], Angélica Fierro[1,4]*

[1]Neurochemistry and Molecular Modeling Lab, Department of Organic Chemistry, Faculty of Chemistry and of Pharmacy, Pontificia Universidad Católica de Chile, Santiago 7820436, Chile.

*E-mail: afierroh@uc.cl

[2]Receptomics and Signaling Networks in Brain Diseases (Group C22), Instituto de Investigación Biomédica de Málaga y Plataforma en Nanomedicina–IBIMA Plataforma BIONAND. 29010 Malaga, Spain.

[3]Receptomics & Brain Disorders Lab, Department of Human Physiology and Physical Education and Sport Sciences, School of Medicine, University of Malaga. 29010 Malaga, Spain.

[4] Centro Interdisciplinario de Neurociencia Aplicada, Pontificia Universidad Católica de Chile. Santiago 7820436, Chile.

Table of Contents

Figure S1: Validation of $D2_SR$, $D2_LR$, and GHSR1a models ……………….…………………… S3

Figure S2: Trajectory analysis of CG-MD simulations of $D2_SR$, $D2_LR$, and GHSR1a …….………..S4

Figure S3: Trajectory analysis of CG-MD simulations of $D2_{S,L}R$/GHSR1a heteromers ……………..S5

Figure S4: $D2_{S,L}R$/GHSR1a heteromers from self-assembly assays.…………………….………….S6

Figure S5: Contact network analysis of self-assembly $D2_{S,L}R$/GHSR1a heteromers…….……………...S7

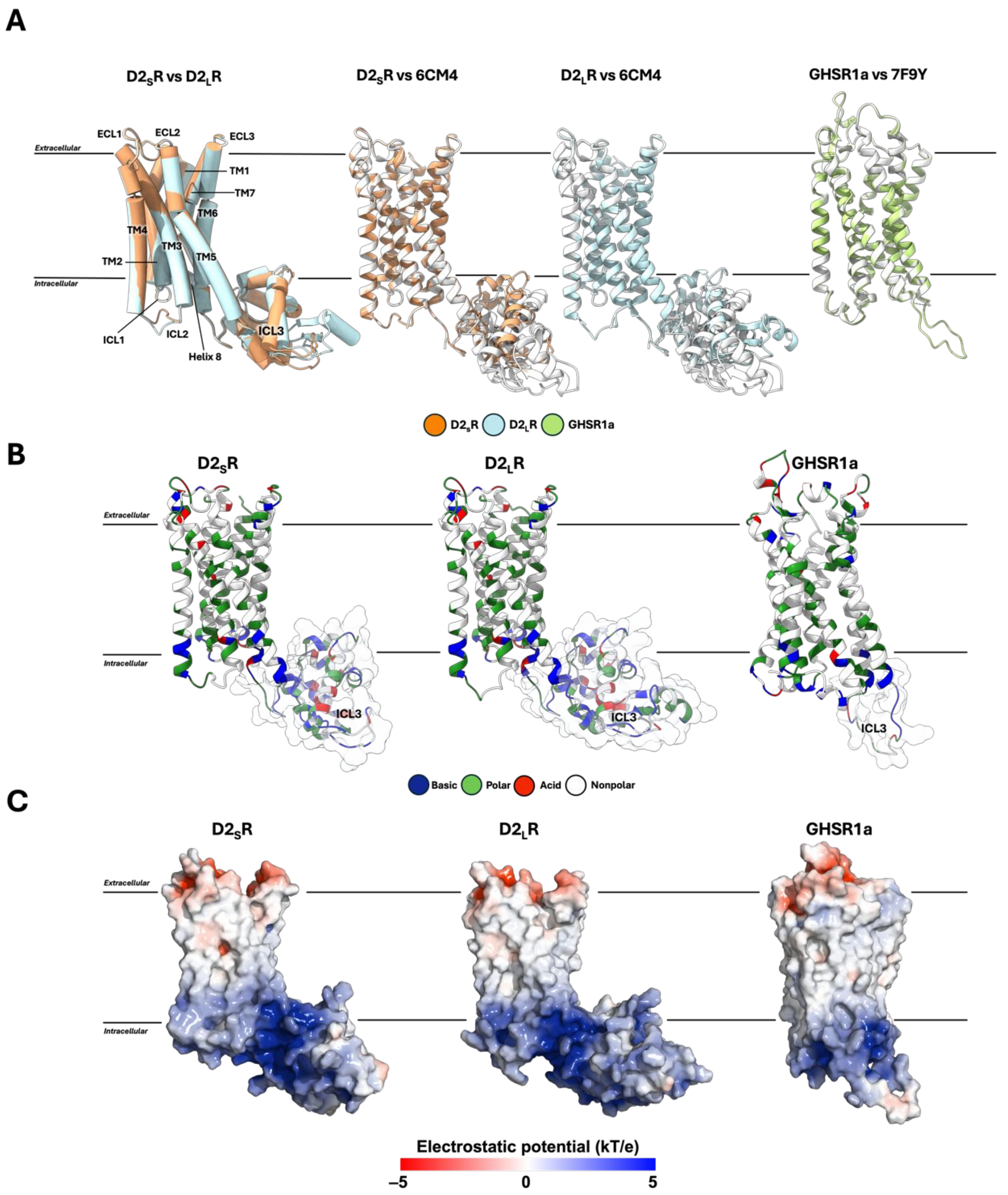

Figure S1. Validation of D2$_S$R, D2$_L$R, and GHSR1a models. A. Models obtained through homology modeling and their superposition with D2R crystallographic structure (PDBid: 6CM4) and GHSR1a crystallographic structure (PDBid: 7F9Y). **B.** Aminoacidic nature of the three models, with charged residues to both extra- and intracellular, and non-charged residues in the membrane. **C.** Adaptative Poisson-Boltzmann Solver (APBS) shows negative electrostatic potential for the orthosteric binding pocket, with a positive potential at the G-protein recognition site intracellularly.

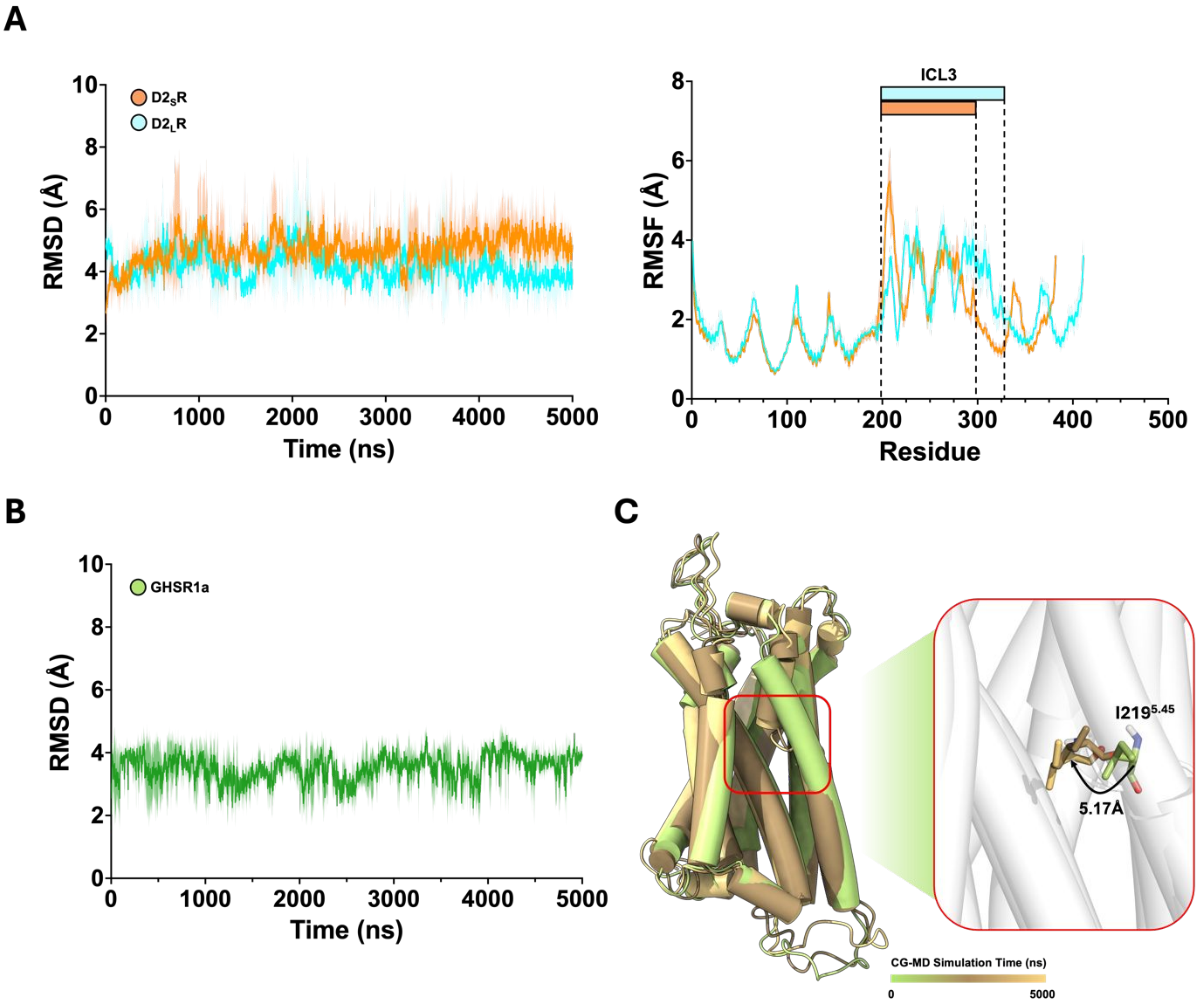

Figure S2. Trajectory analysis of CG-MD simulations of $D2_SR$, $D2_LR$, and GHSR1a. A. RMSD and RMSF analysis of $D2_SR$ (orange) and $D2_LR$ (cyan) through the 5µs CG-MD simulations (n=3, 15µs total). In the RMSF plot, ICL3 fluctuation and size differences are highlighted. **B.** RMSD analysis of GHSR1a (green) through the 5µs CG-MD simulations (n=3, 15µs total). **C.** Three frames (initial, middle, and end frame) from one of the 5µs CG-MD simulations. I219$^{5.45}$ side chain fluctuation is amplified within the red box.

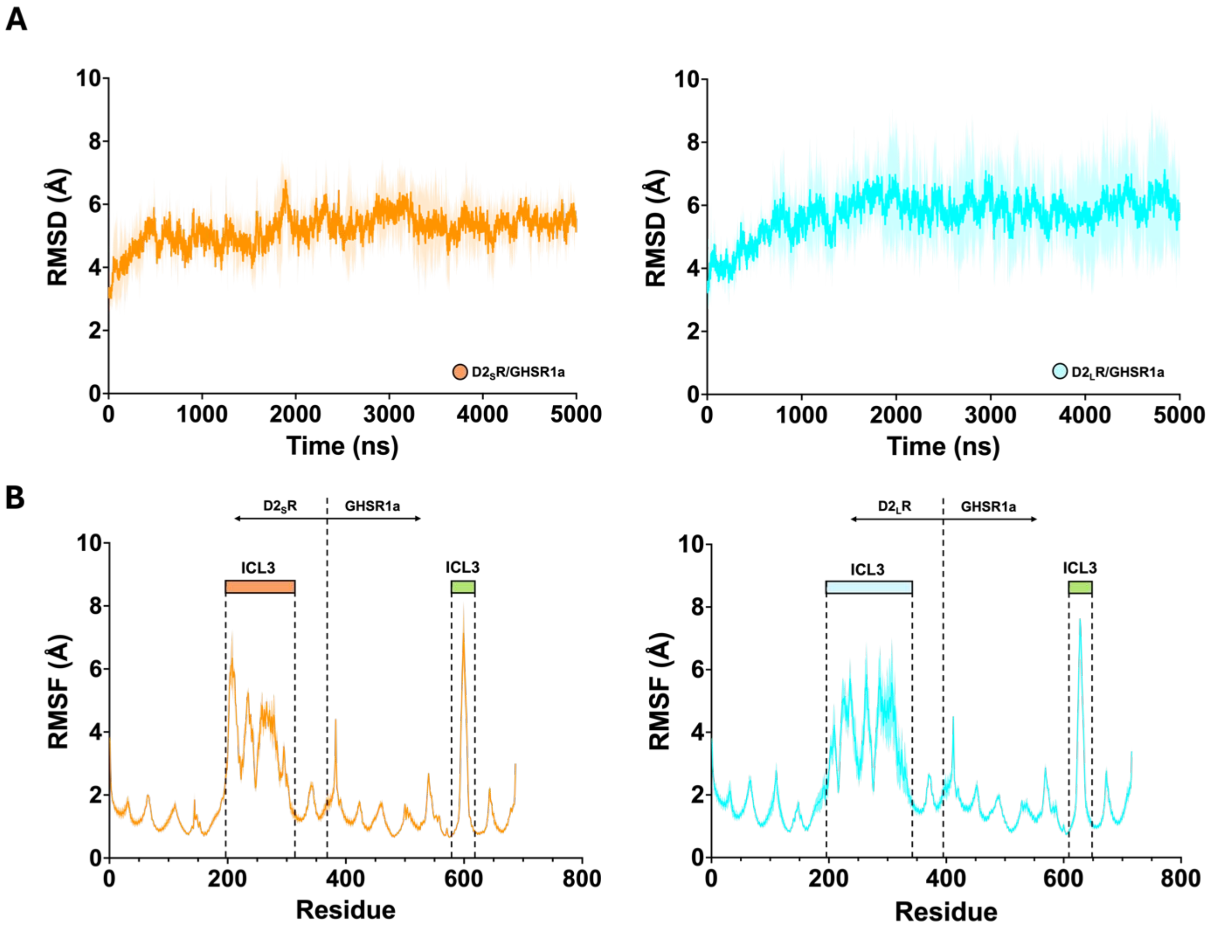

Figure S3. Trajectory analysis of CG-MD simulations of D2$_{S,L}$R/GHSR1a heteromers. A. RMSD analysis of D2$_S$R/GHSR1a (orange) and D2$_L$R/GHSR1a (cyan) heteromers generated with HADDOCK through the 5μs CG-MD simulations (n=3, 15μs total). **B.** RMSF analysis of D2$_S$R/GHSR1a (orange) and D2$_L$R/GHSR1a (cyan) heteromers generated with HADDOCK through the 5μs CG-MD simulations (n=3, 15μs total). Both receptors are plotted together, with their respective ICL3 fluctuations highlighted.

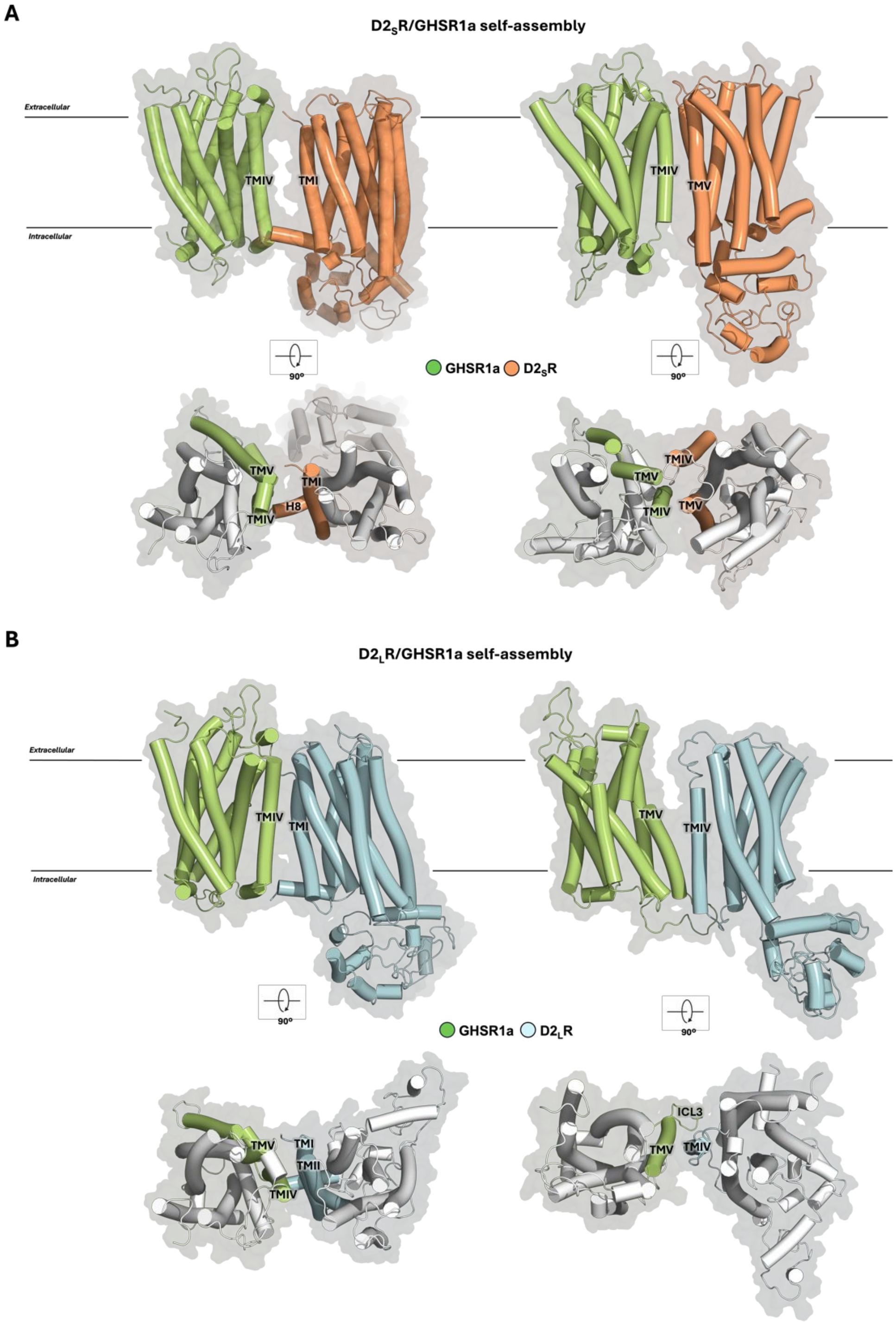

Figure S4. $D2_{S,L}R$/GHSR1a heteromers from self-assembly assays. A. Backmapped $D2_SR$/GHSR1a all-atom heteromers with two different interaction interfaces, TMI/TMIV (left) and TMV/TMIV (right). **B.** Backmapped $D2_LR$/GHSR1a all-atom heteromers with two different interaction interfaces, TMI/TMIV (left) and TMIV/TMV (right).

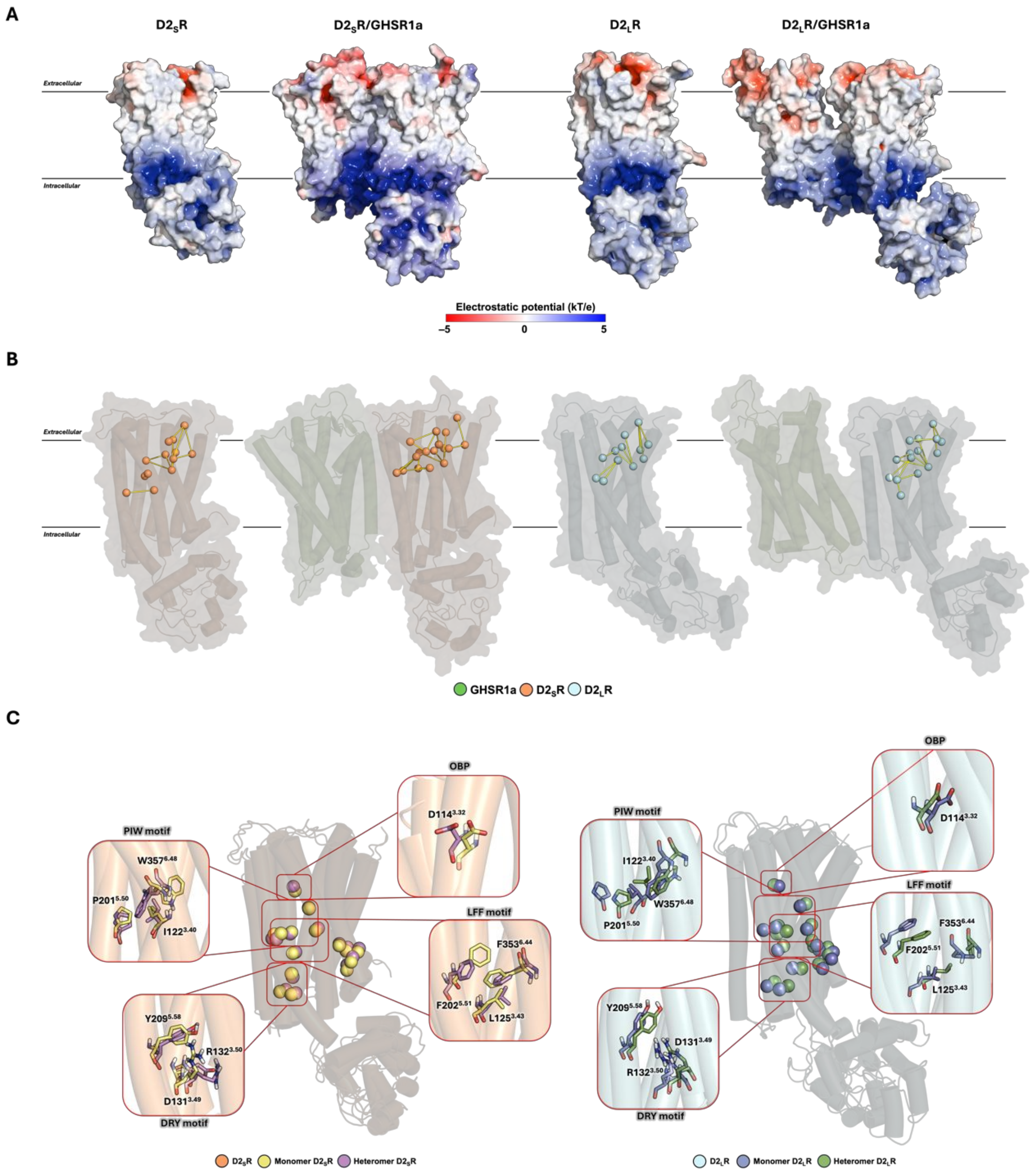

Figure S5. Contact network analysis of self-assembly $D2_{S,L}R$/GHSR1a heteromers. A. APBS shows negative electrostatic potential for D2R isoform orthosteric binding pockets as monomers and heteromers. **B.** OBP's contact network for D2R isoforms as monomers and heteromers. Cα atoms in spheres as main residues potentially interacting with ligands. **C.** Main activation residues change analysis was structured around the determinant residues associated to the $D114^{3.32}$ as the main ligand-protein interaction at the OBP, a "PIW" motif ($P^{5.50}$, $I^{3.40}$, and $W^{6.48}$), "LFF" motif ($L^{3.43}$, $F^{5.51}$, and $F^{6.44}$), and the "DRY" motif ($D^{3.49}$, $R^{3.50}$, $Y^{5.58}$).
